# Resequencing the Yaroslavl cattle genomes reveals signatures of selection and a rare haplotype on BTA28 likely to be related to breed phenotypes

**DOI:** 10.1111/age.13230

**Published:** 2022-06-16

**Authors:** Daniil Ruvinskiy, Alexander Igoshin, Andrey Yurchenko, Anna V. Ilina, Denis M. Larkin

**Affiliations:** ^1^ The Federal Research Center Institute of Cytology and Genetics Siberian Branch of the Russian Academy of Sciences (ICG SB RAS) Novosibirsk Russia; ^2^ Kurchatov Genomics Center, Institute of Cytology and Genetics Siberian Branch of the Russian Academy of Sciences Novosibirsk Russia; ^3^ Federal Williams Research Center of Forage Production & Agroecology Scientific Research Institute of Livestock Breeding and Forage Production Yaroslavl Region Russia; ^4^ Royal Veterinary College University of London London UK

**Keywords:** 1000 Bull Genomes Project, *F*_ST_, hapFLK, *KAT6B*, signatures of selection, Yaroslavl cattle

## Abstract

The genomes of local livestock could shed light on their genetic history, mechanisms of adaptations to environments and unique genetics. Herein we look into the genetics and adaptations of the Russian native dairy Yaroslavl cattle breed using 22 resequenced individuals and comparing them with two related breeds (Russian Kholmogory and Holstein), and to the taurine set of the 1000 Bull Genomes Project (Run 9). HapFLK analysis with Kholmogory and Holstein breeds (using Yakut cattle as outgroup) resulted in 22 regions under selection (*q*‐value < 0.01) on 11 chromosomes assigned to Yaroslavl cattle, including a strong signature of selection in the region of the *KIT* gene on BTA6. The *F*_ST_ (fixation index) with the 1000 Bull Genomes Dataset showed 48 non‐overlapping top (0.1%) *F*_ST_ regions of which three overlapped HapFLK regions. We identified 1982 highly differentiated (*F*_ST_ > 0.40) missense mutations in the Yaroslavl genomes. These genes were enriched in the epidermal growth factor and calcium‐binding functional categories. The top *F*_ST_ intervals contained eight genes with allele frequencies quite different between the Yaroslavl and Kholmogory breeds and the rest of the 1000 Bull Genomes Dataset, including *KAT6B*, which had a nearly Yaroslavl breed‐specific deleterious missense mutation with the highest *F*_ST_ in our dataset (0.99). This gene is a part of a long haplotype containing other genes from *F*_ST_ and hapFLK analyses and with a negative association with weight and carcass traits according to the genotyping of 30 phenotyped Yaroslavl cattle individuals. Our work provides the industry with candidate genetic variants to be focused on in breed improvement efforts.

Dwelling in harsh climate conditions, Russian cattle breeds have been required to develop adaptations which can now be traced in the genome regions under selection and convergently evolving genes (Buggiotti et al., 2021). One of the prominent dairy cattle breeds formed in the European part of Russia from the seventeenth century is the Yaroslavl cattle. This breed originated from Northern Great Russian cattle, which were small in stature and low in productivity, but able to survive in harsh environments with poor foraging opportunities (Liskun, 1949). The resulting Yaroslavl breed is adaptable, feed efficient and maintains good reproductive ability even at low temperatures with a lack of forage, which made it especially popular among peasants (Liskun, 1919). The Yaroslavl breed is black, with a white stomach, legs, tail and head, with black markings resembling ‘glasses’. The aim of this study was to identify the signatures of selection and genetic variants contributing to the phenotypes and adaptations of Yaroslavl cattle. Studying breeds such as the Yaroslavl is important for conservation purposes, breeding in a sustainable and efficient manner and for the genetic improvement of locally adapted livestock.

Twenty‐two Yaroslavl cattle individuals were selected for this study. Samples were resequenced using Illumina’s Hiseq4000 technology at Novogene (HK) Co. Ltd (China) to ~50 Gbp each. Cleaned reads were mapped to the reference cattle genome assembly (bosTau9) using BWA‐MEM and processed using the 1000 Bull Genomes Project pipeline (gatk v. 3.8; https://gatk.broadinstitute.org/hc/en‐us). GVCF files were submitted to the 1000 Bull Genomes Project and included in Run 9. After quality control (QC) and linkage disequilibrium pruning, we calculated the pairwise PI_HAT measure (proportion IBD, that is, *P*(IBD = 2) + 0.5*P(IBD = 1)) of relatedness using plink (−genome) software (Table S1).

Of 22 Yaroslavl cattle individuals, the 10 purest animals based on their structure and principal component analysis results (Yurchenko et al., 2018) were selected for a signatures of selection scan with hapFLK (Fariello et al., 2013). These samples were mapped against bosTau6 following the protocol described in Buggiotti et al. (2021) and merged with the pre‐existing GVCF files of Kholmogory (20), Yakut (20) and Holstein (20) breed individuals (Buggiotti et al., 2021) to obtain a joint vcf file (see Appendix S1 for breed descriptions). Variant calling was done following the genome analysis toolkit (gatk; Van der Auwera et al., 2020) pipeline. Filtering of single nucleotide polymorphisms (SNPs) for quality (‘hard filtering’) was applied using the following parameters: (i) variant confidence/quality by depth < 2; (ii) RMS mapping quality (MQ) < 40.0; (iii) Phred‐scaled *p*‐value using Fisher’s exact test to detect strand bias > 60; (iv) *Z*‐score from the Wilcoxon rank sum test of alternative vs. reference read MQs (MQRankSum) <−12.5; and (v) *Z*‐score from the Wilcoxon rank sum test of alternative vs. reference read position bias (ReadPosRankSum) <−8. The thresholds for these parameters were adopted from gatk Best Practices (Van der Auwera et al., 2020). Indel variants were removed. Multiallelic SNPs were converted to biallelic format using bcftools with *norm ‐m* parameters. The resulting VCF file was converted to plink format removing individuals with call rate < 0.09 and markers with missingness > 0.01 or minor allele frequency (MAF) < 0.1 for the signatures of selection scan with hapFLK (‐K 30 ‐‐nfit = 30) with the Yakut cattle samples as an outgroup. The *p*‐values were calculated using the scaling_chi2_hapflk.py script (Fariello et al., 2013). *Q*‐values were calculated using the ‘qvalue’ R function (Storey & Tibshirani, 2003). Weighted *F*_ST_ (fixation index) statistics were calculated for 22 Yaroslavl cattle individuals against 4 779 animals (coverage >6×) of taurine cattle from the 1000 Bull Genomes Project (Run 9) using vcftools v.0.1.13 with the parameters ‐‐fst‐window‐size 50 000 ‐‐fst‐window‐step 25 000 ‐‐max‐missing 0.9 and for individual SNPs omitting the ‐‐fst ‐window‐size, ‐‐fst‐window‐step options. Translation of the cattle genome coordinates between the UMD3.1 and ARS/UCD1.2 builds was performed using the UCSC Genome Browser *liftover* tool with default parameters. The top 0.1% *F*_ST_ windows were compared with hapFLK intervals. The direction of selection was determined using local phylogenetic trees and haplotype clustering for hapFLK intervals with no *F*_ST_ interval overlaps. Candidate SNPs were annotated with the NGS‐SNP pipeline (Grant et al., 2011; bosTau6) and snpeff (Cingolani et al., 2012; bosTau9). Missense variants within the genes *MSS51* and *KAT6B* were amplified using 30 Yaroslavl phenotyped cattle individuals with PCR (Appendix S2). The PCR products were cut with HinfI and HpySE526 I restriction enzymes (SibEnzyme Ltd) for *MSS51* and *KAT6B* products, respectively. The association between phenotypes obtained from individual dam record cards and genotypes (Table S2) was estimated using one‐way ANOVA (‘aov’ R function) and linear regression (‘lm’ R function).

A total of 27 391 619 SNPs were identified in the set of four breeds (Yaroslavl, Kholmogory, Holstein, and Yakut), of which 6 069 156 were used in the hapFLK analysis. This resulted in 22 regions under selection (*q*‐value < 0.01) on 11 chromosomes assigned to Yaroslavl cattle (Figure 1; Table S3). A comparison of Yaroslavl individuals against the 1000 Bull Genomes Dataset resulted in 48 non‐overlapping regions with the top 0.1% of *F*_ST_ values (Figure S1; Table S4). Four intervals had overlaps between the hapFLK and *F*_ST_ results, of which one (BTA29: 27 527 228‐27 711 900) contained a single gene, *TMEM225*, with one missense mutation (Phe73Leu) showing an *F*_ST_ value of 0.76 when Yaroslavl cattle were contrasted against the 1000 Bull Genome Dataset. Transmembrane protein 225B was previously related to hyperactivity of sperm in pigs (van Son et al., 2020) and therefore could contribute to fertility traits. HapFLK revealed a strong signature of selection on BTA6: 71 464 554‐71 892 219 containing *KIT*, which is known to be a ‘spotting’ gene in cattle (Fontanesi et al., 2010). We did not identify any missense mutations with high *F*_ST_ in this region, suggesting that the selection acted on haplotypes and regulatory sequences. Eight additional genes were found in intervals with the top *F*_ST_ values and had missense mutations with derived allele frequencies quite different between the Yaroslavl cattle, Kholmogory, and the rest of the taurine 1000 Bull Genomes Dataset. These genes included *RHBDL2*, *FCHO1*, *LPIN1*, *TTC28*, *SNX25*, *KAT6B*, *VRK2* and *ZNF239*. Of these, *SNX25* contained five missense mutations with high *F*_ST_ (*F*_ST_ range 0.53–0.56) and *TTC28* contained four (*F*_ST_ range 0.95–0.97). *SNX25* is a key gene in the regulation of TGF‐β signalling, and therefore contributes to the immune system (Nishimura et al., 2021), whereas *TTC28* is important for spindle assembly in mitosis and meiosis (Chen et al., 2018). The highest missense mutation *F*_ST_ value was observed for the KAT6B, Val105Met variant (*F*_ST_ = 0.993), the highest among all of the Yaroslavl cattle missense variants, suggesting that this variant is nearly Yaroslavl cattle specific. Indeed, among 5 074 remaining animals from the taurine set of the 1000 Bull Genomes Project successfully genotyped at this locus, the same mutation was found only in a heterozygous state in two Modern Angler and two Lithuanian Red individuals while the frequency of the derived A allele was 0.64 in the resequenced Yaroslavl cattle population. A 50 kb region with high *F*_ST_ (0.36) in Yaroslavl cattle also contained *KAT6B*. This interval was 574 kbp away from a strong signature of selection identified by the hapFLK and containing genes *VCL* and *AP3M1*. In between them another 200 kbp region with *F*_ST_ = 0.53 was located containing *ADP*. An additional missense mutation with high *F*_ST_ (0.986) was located upstream of *VCL* in *MSS51*. PCR genotyping and association analysis of *MSS51* and *KAT6B* mutations using 30 Yaroslavl cattle individuals revealed the complete linkage of derived alleles and a negative association of the haplotype with weight and carcass traits (Table S5 and Figure S2). When the haplotype structure of the region between *MSS51* and *KAT6B* was examined, it indeed showed a long haplotype present only in the Yaroslavl individuals (Figure 2). *F*_ST_ and hapFLK data suggest multiple signatures of selection in this region or alternatively the hitchhiking of some mutations within the selected haplotype. The NGS‐SNP analysis of the *MSS51* and *KAT6B* missense variants suggests a deleterious effect of the KAT6B substitution (sorting intolerant from tolerant, SIFT = 0.01; Table S6) and high evolutionary conservation of the reference genome amino acid, which was found in this position in most mammals checked on the NCBI protein homology database (Table S6). The change observed is found in a highly conserved histone H15 domain, further supporting its functional effect. Genes within this haplotype have functions relevant to economically important traits explaining possibilities for single or multiple independent selection signals. MSS51 is a skeletal muscle‐specific protein involved in fibre type determination and metabolism (Moyer & Wagner, 2015). *ADK* was previously found under selection in Simmental cattle and was linked to the physiological state of animals (Ramey et al., 2013). VCL is a cytoskeletal protein involved in anchoring F‐actin to membranes. Defects in VCL lead to dilated cardiomyopathy (Wells et al., 2011) similarly to NRAP, recently found evolving convergently in cold‐adapted Yakut cattle and hibernating and cold‐adapted species (Buggiotti et al., 2021). *KAT6B* was previously associated with carcass traits including leg morphology in cattle (van den Berg et al., 2014). Our data and the results of the association analysis suggest that the Yaroslavl haplotype is responsible for lower weight and height, which could historically be beneficial under the harsh environmental conditions and low feeding base in Russia. The lack of this haplotype in most other cattle breeds suggests its negative effect in other environmental conditions and/or negative selection by humans. This implies that the breeds that still have derived alleles in low frequencies (e.g. Angler and Lithuanian Red) could potentially benefit from further elimination of this haplotype.

**FIGURE 1:**
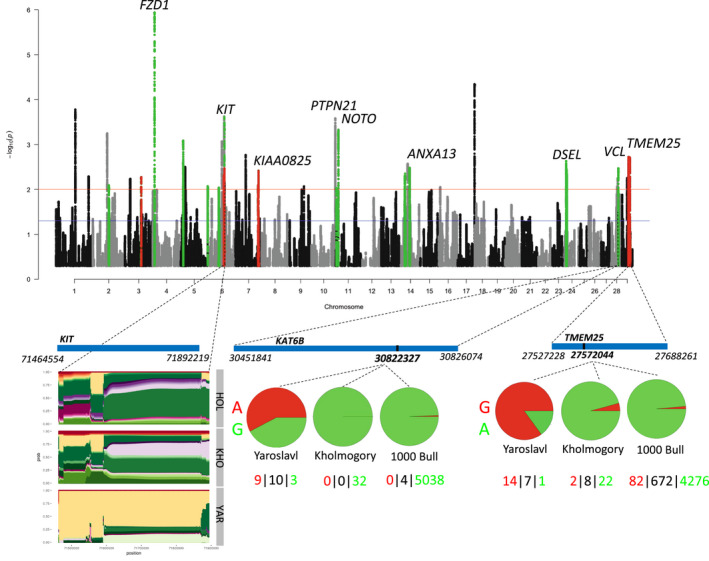
HapFLK results for the analysis of Yaroslavl, Kholmogory, and Holstein breeds. In green are regions under selection in Yaroslavl cattle. The blue line shows the significance at *q*‐value = 0.05, while the red line shows *q*‐value = 0.01. In red are regions overlapping between the hapFLK and *F*_ST_ analyses. The region under selection overlapping the *KIT* gene is shown with haplotype diversity for Holstein (HOL), Kholmogory (KHO), and Yaroslavl (YAR) cattle. The positions of missense mutations are shown for the *KAT6B* and *TMEM25* genes with the number of homozygous for both alleles and heterozygous individuals indicated below the frequency chats. Green indicates the reference allele while the red indicates the derived allele

**FIGURE 2:**
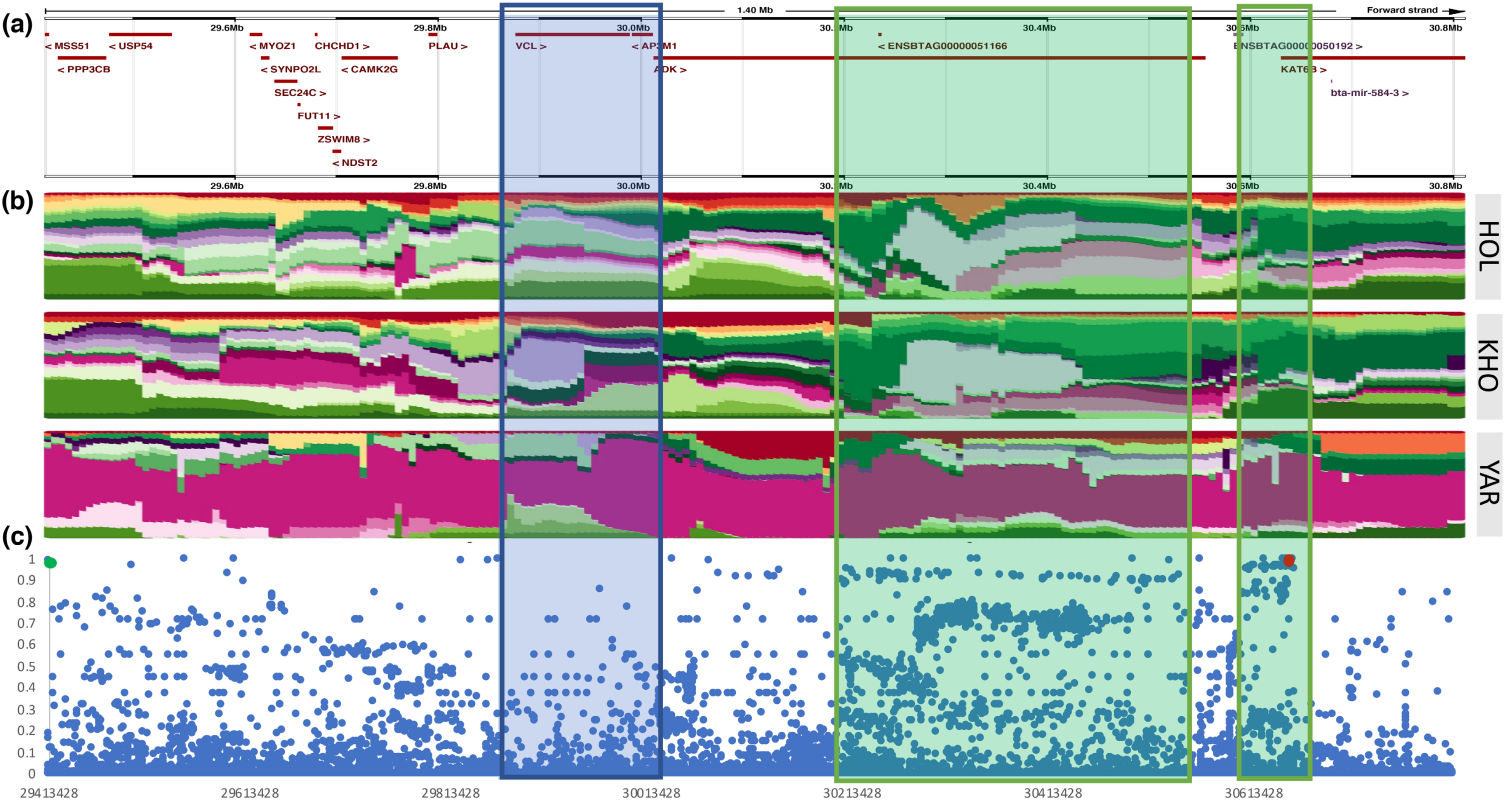
A 1.4 Mbp interval on BTA28 between *MSS51* and *KAT6B*. (a) gene track from ensembl. (b) Haplotypes in Holstein, Kholmogory, and Yaroslavl breeds with the Yaroslavl‐specific haplotype shown in purple. The hapFLK interval under selection, containing *VCL* and *AP3M1* genes, is shadowed in blue. In green are shadowed two intervals with high *F*_ST_ values. (c) Yaroslavl cattle SNP‐based *F*_ST_ analysis with the 1000 Bull Genomes Project Dataset, indicating the positions of the *MSS51* missense mutation in green and the *KAT6B* missense mutation in red

Overall, we found 1 981 high‐frequency missense variants (Yaroslavl cattle MAF >0.1) with SNP *F*_ST_ values >0.4 when compared with the rest of the taurine animals from the 1000 Bull Genomes Project (Table S6). These SNPs were found in 1 483 genes. david functional clustering analysis (Table S7) revealed 16 clusters (enrichment score > 1.3), of which the top cluster (enrichment score = 4.87) contained the functional categories *epidermal growth factor* (false discovery rate, FDR = 0.0004), *epidermal growth factor‐like domain* (FDR = 0.0004), *EGF‐like calcium‐binding* (FDR = 0.001), etc. The epidermal growth factor plays an important role in mammary gland development and epithelial regeneration and contributes to innate immune responses (Fu et al., 2015; Gabadage et al., 2017), suggesting that genes from this cluster could contribute to the milk production traits. Another functional cluster (enrichment score = 1.87) contained multiple terms related to *keratin*, suggesting that keratin genes have evolved in Yaroslavl cattle, probably providing extra protection to animals during the long and cold winters of Russia.

Our work provides the first nucleotide‐level genome‐wide assessment of the Russian Yaroslavl cattle genomes in the context of breeds from around the world and points to signatures of selection and specific genetic variants which could contribute to adaptations and phenotypes for further breed improvement.

## Supporting information


Appendix S1

Appendix S2

Figure S1
Figure S2

Table S1
Table S2
Table S3
Table S4
Table S5
Table S6
Table S7

## Data Availability

Twenty‐two Yaroslavl cattle resequenced genomes were submitted to NCBI SRA and are available under the accession number PRJNA814817.
