# Supplementary material for: Resequencing the Yaroslavl cattle genomes reveals signatures of selection and a rare haplotype on BTA28 likely to be related to breed phenotypes
Source: Anim Genet. 2022 Jun 16;53(5):680–4. doi: 10.1111/age.13230 (PMC9541747; doi:10.1111/age.13230)
Supplement: Supplementary file 1 — Appendix S1 [file AGE-53-680-s001.docx]

**Breed descriptions**

**
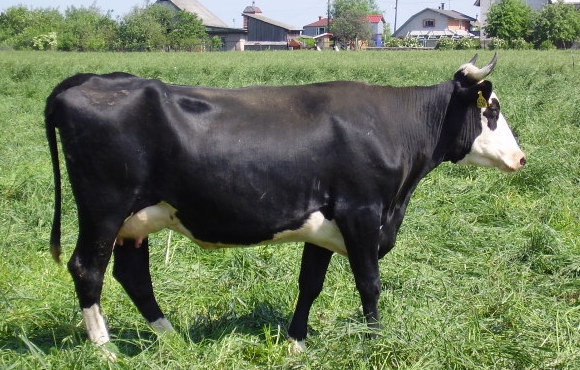
**

**Yaroslavl cattle**. This dairy breed was established in 19th century for its high degree of adaption to the conditions of the Yaroslavl region of Russia, where it hails from. With a unique spotting pattern resembling a black “mask” on their otherwise white head, the average representative of the breed stands at a height of 125-127 cm and a weight of 450-500 kg for cows and 700-800 kg for bulls. Their milk is well suited for cheesemaking, and actively used in traditional cheeses particular to the region. In addition, these cattle are considered to be fairly resistant to tuberculosis and brucellosis. At this moment, this breed is in dramatic decline (from close to a million in the 1960s to around 50 thousand in 2015), due to competition and crossbreeding with imported high output dairy cattle.


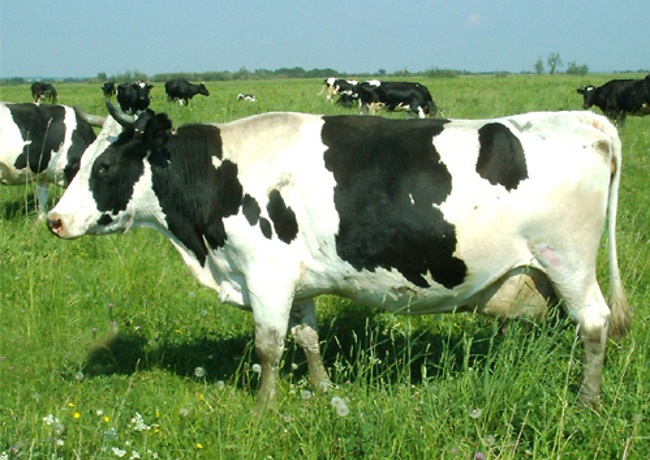


**Kholmogory cattle.** This breed is considered to be one of the oldest breeds in Russia, with mentions of it’s milk quality and export to other regions dating back as early as the 17^th^ century. This breed is from the Kholmogory and Archangelsk region and has black or red and white coat pattern. Through meticulous artificial selection, breed and parlour management this breed was not only selected for its adaptation to the local region and its alluvial flood plains, but also its high milk yield, hardiness and disease resistance. With a height of 133-135 cm and a weight of 570-590 kg for cows and up to 1170 kg for bulls, this breed has a long history of crossing with outside breeds from Holland and a population of over 2 million heads.

**
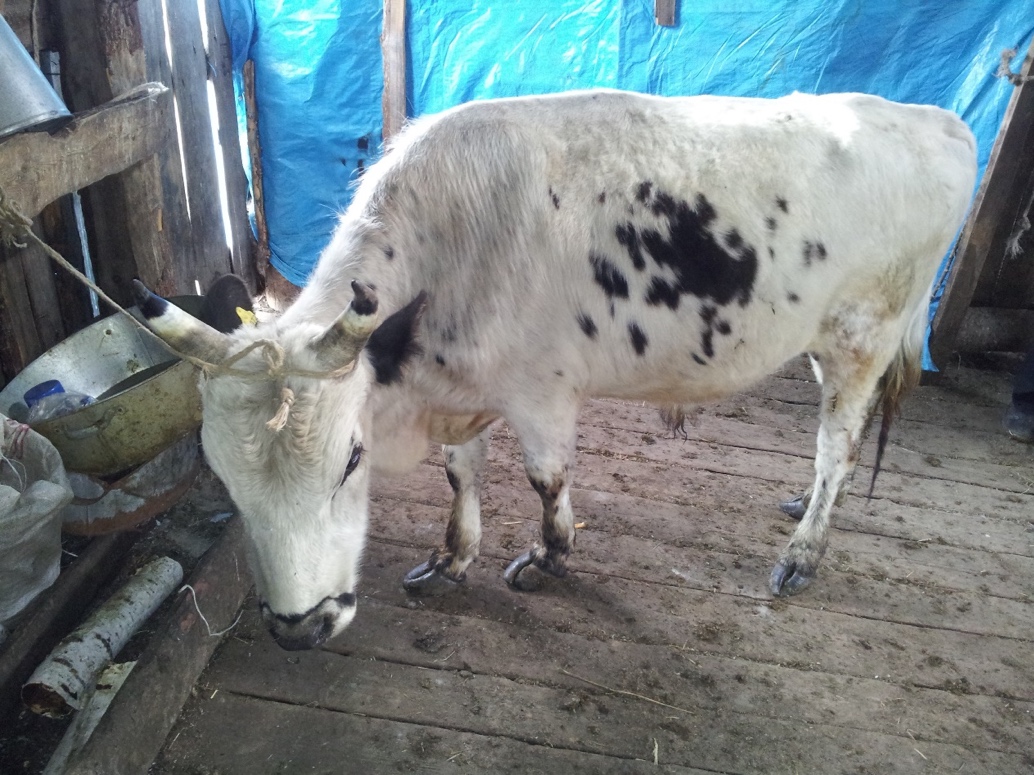
**

**Yakut cattle.** This is a hardy breed from the republic of Sakha (Yakutia) that has adapted perfectly to survival above the arctic circle. To deal with Siberia’s harsh climate and sparse food sources, this breed has developed thermoregulatory and metabolic adaptations that allow it to graze all year round in temperatures as low as -50C. Due to being present in a remote and isolated habitat this breed has been genetically preserved and not been frequently crossed with industrial cattle. This is a small, stocky breed with a height of 110-112 cm and a weight of 350-400 kg for cows and 500-600 kg for bulls. This breed is under threat of extinction with a population decline from 494 thousand heads in 1928 to only ~1500 individuals now.
