# Supplementary material for: Resequencing the Yaroslavl cattle genomes reveals signatures of selection and a rare haplotype on BTA28 likely to be related to breed phenotypes
Source: Anim Genet. 2022 Jun 16;53(5):680–4. doi: 10.1111/age.13230 (PMC9541747; doi:10.1111/age.13230)
Supplement: Supplementary file 2 — Appendix S2 [file AGE-53-680-s004.docx]

**Appendix S1.** Primers and PCR conditions for the analysis of *MSS51* and *KAT6B* alleles.

G>A *KAT6B* substitution (Val105Met):

Forward primer: ACTTGCAAACCCACTTTATACAGAGTGG

Reverse primer: CTGATCTTTCTCGTGGGGTAGAAGG

1 cycle:

95 °C – 3 min

35 cycles:

95 °C – 1 min

60 °C – 1 min

72 °C – 55 sec

Amplicon length: 613 bp, upon HpySE526 I digestion: 303 bp and 310 bp, cleavage at the presence of ancestral (G) allele

G>T *MSS51* mutation (Ala415Glu):

Forward primer: CTTGGCTTTCTTATCCCTTCAAAGTGC

Reverse primer: ATCCAGTCATGATCTGGCTCAGC

1 cycle:

95 °C – 3 min

35 cycles:

95 °C – 1 min

58 °C – 1 min

72 °C – 45 sec

Amplicon length: 390 bp, upon HinfI digestion: 224 bp and 166 bp, cleavage at the presence of Yaroslavl-specific (T) allele
