# Supplementary material for: Resequencing the Yaroslavl cattle genomes reveals signatures of selection and a rare haplotype on BTA28 likely to be related to breed phenotypes
Source: Anim Genet. 2022 Jun 16;53(5):680–4. doi: 10.1111/age.13230 (PMC9541747; doi:10.1111/age.13230)
Supplement: Supplementary file 3 — Figure S1 Figure S2 [file AGE-53-680-s002.pdf]

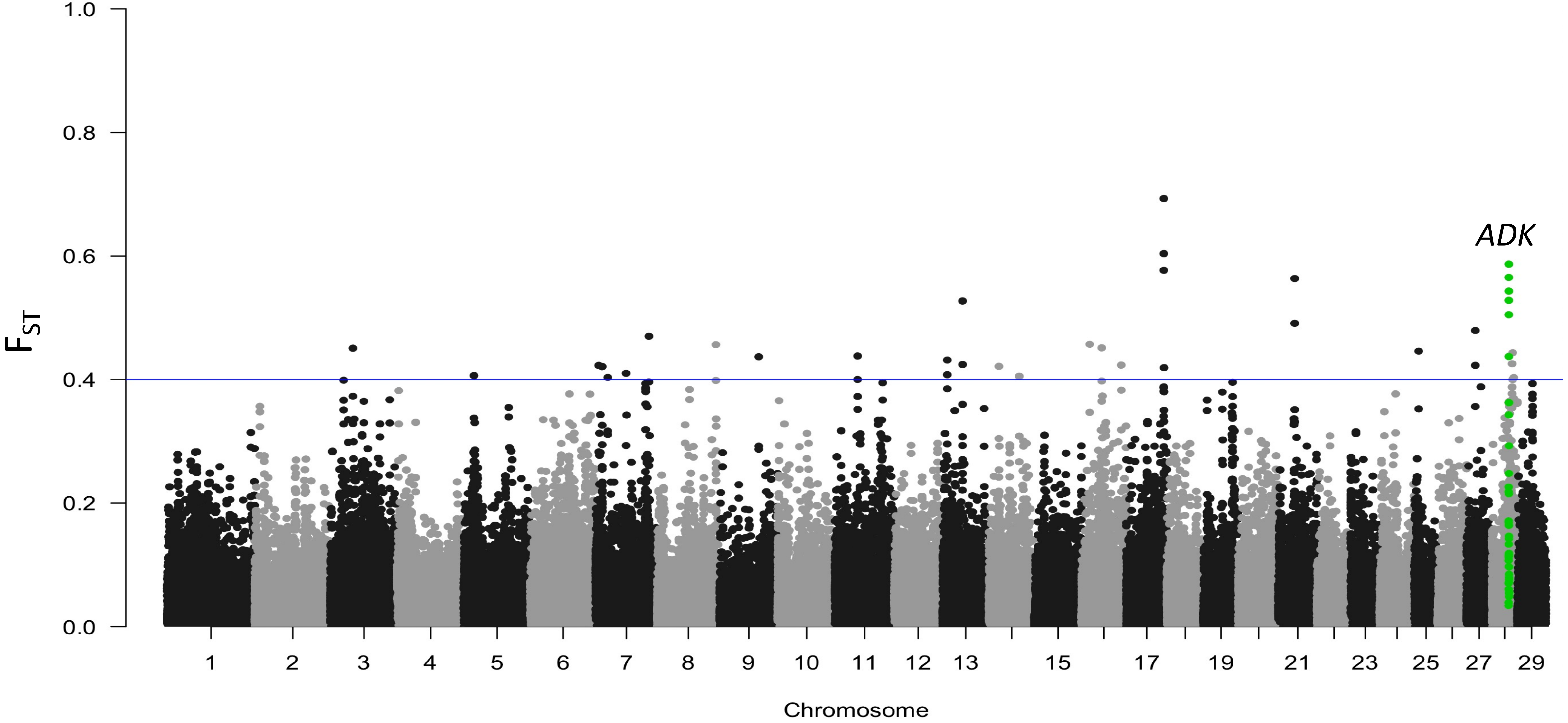

Figure S1. F_{ST} results for 22 *Yaroslavl* cattle samples (50K windows) compared to ~5K taurine cattle individuals from the 1K Bull Project. In green is the region on BTA28, containing two high-frequency Yaroslavl cattle missense mutations in *MSS51* and *KAT6B*. Blue line shows $F_{ST} = 0.4$.

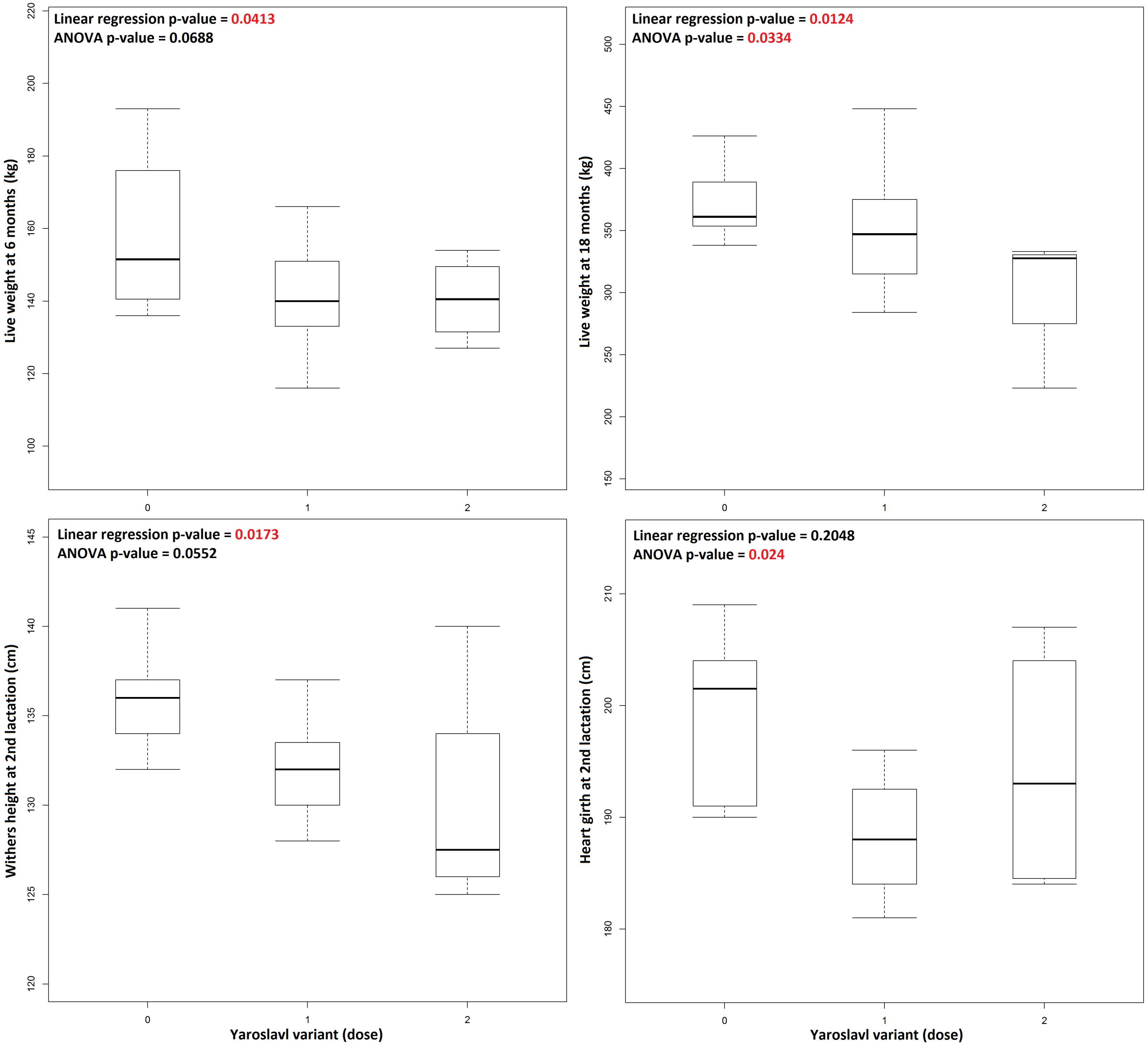

Figure S2. Phenotypes according to *MSS51/KAT6B* mutation doses. 0 = ancestral amino acid; 1 = heterozygous for Yaroslavl allele; 2 = homozygous for Yaroslavl allele.
